# The Cultural Diversity Climate “Big Five” in Schools: Dimensions, Facets, and a Future Research Agenda

**DOI:** 10.1177/10888683261458924

**Published:** 2026-07-03

**Authors:** Lisa Bardach, Miriam Schwarzenthal

**Affiliations:** 1 Department of Psychology, University of Giessen, Germany; 2 Kaunas University of Technology, Lithuania; 3 School of Education, University of Wuppertal, Germany

**Keywords:** diversity climate, intergroup contact theory, multiculturalism, colorblind, critical consciousness, polyculturalism

## Abstract

**Academic Abstract:**

Children and adolescents are growing up in increasingly ethnically, racially, and culturally diverse and often unequal worlds. Therefore, how schools, as key developmental contexts, approach diversity has important implications at individual, group, and societal levels. Here, we present a review, integration, and expansion of research on cultural diversity climate in schools, focusing on five dimensions of diversity climate (the cultural diversity climate “Big Five”) and their facets. We begin by distinguishing cultural diversity climate from other related terms (e.g., diversity ideologies, diversity policies). Then, we discuss the five core dimensions of diversity climate (intergroup contact theory’s optimal contact conditions, multiculturalism climate, colorblind climate, critical consciousness climate, polyculturalism climate). We examine facets subsumed under the dimensions, propose new theory-based facets, and outline differences and commonalities between them. Next, we review measurement approaches and highlight future research directions. Overall, this integrative, multidimensional, and multifaceted framework advances psychological theory on diversity climate.

**Public Abstract:**

Schools play a vital role in how children and adolescents experience and understand diversity. This article reviews the concept of cultural diversity climate in schools, focusing on five key aspects that shape how cultural diversity is approached (i.e., the cultural diversity climate “Big Five”). Our integration of the cultural diversity climate “Big Five” encompasses five main dimensions, such as multiculturalism climate (acknowledging and valuing diversity at school), critical consciousness climate (critically reflecting on and challenging racism and inequities), and polyculturalism climate (raising students’ awareness of connections between groups and cultures). To move towards a more fine-grained understanding of diversity climate, we describe existing facets (i.e., sub-components of the different dimensions) and develop new facets based on theory. We also discuss different ways of measuring cultural diversity climate. This work is important for creating more inclusive, equitable, and critically conscious school environments, thereby supporting all students, especially those from marginalized communities.

## Introduction

Due to globalization, immigration, and demographic shifts, increased diversity has become a reality of social life in many societies (OECD, 2020; Rosenthal et al., 2019; U.S. Census Bureau, 2019). Diversity can enrich individuals’ lives, foster innovation, and provide unique opportunities for engaging in intercultural interactions (Hundschell et al., 2022; Pettigrew & Tropp, 2006). Positive intercultural interactions, in turn, are a major driver of intercultural learning experiences and cross-group friendships, and contribute to the well-being of ethnic, racial, and cultural majority and minority group members (Leslie et al., 2020; OECD, 2020; Rivas-Drake et al., 2014; Suárez-Orozco et al., 2018). Yet, diversity can also go along with prejudice and intergroup tension (Hewstone, 2015; Leslie & Flynn, 2024). Relatedly, persistent structural and interpersonal discrimination, stigmatization of marginalized groups, and systemic racism and oppression remain pressing concerns in many societies (Axt & To, 2025; Heberle, Rapa, & Farago, 2020; Roberts & Rizzo, 2020; Rosenthal et al., 2019; Salter et al., 2018; Skinner-Dorkenoo et al., 2023).

Therefore, it is necessary to ask how to best leverage the positive effects of diversity and promote inclusion while counteracting the potential negative side effects (e.g., prejudice and discrimination) of increased diversity. One answer to this question lies in how diversity is approached in social environments (i.e., in the *cultural diversity climate*). Cultural diversity climate manifests in the practices, policies, norms, and general atmosphere of social environments and shapes the interactions that take place between individuals in these social environments (e.g., Abacioglu et al., 2019; Byrd, 2017; Phalet & Baysu, 2020; Schachner, 2019; Verkuyten & Thijs, 2013).

Even though a variety of social environments (e.g., workplace, university) can be characterized by their cultural diversity climates, this manuscript focuses primarily on the cultural diversity climate in school settings. Schools are critical developmental contexts for individuals growing up in increasingly diverse societies (Rosenthal et al., 2019). Children and adolescents spend a considerable amount of time at school. Most importantly, schools often offer opportunities for intergroup contact and provide a regulatory context for structuring intergroup relations, as well as valuable learning opportunities to explore the history and traditions of one’s own and other students’ cultural, ethnic, and racial groups (Aldana & Byrd, 2015; Tropp et al., 2016). Over time, school-aged children develop a more sophisticated understanding of diversity (especially in adolescence), which provides a fertile foundation for attempts to reduce negative intergroup attitudes (Brown, 2017; Killen et al., 2022; Quintana, 1998; Raabe & Beelmann, 2011).

There is a long tradition of examining cultural diversity climate in schools (Green et al., 1988; Molina & Wittig, 2006; Schachner, Schwarzenthal, Moffitt, et al., 2021; Tropp et al., 2022). Prior research has supported the importance of cultural diversity climate at school, as indicated by its relationships with a range of outcomes relevant to child and youth development. These previously investigated outcomes include intergroup attitudes and relations (e.g., prejudice, discrimination, Baysu et al., 2016; Benner & Graham, 2013; Schachner, Schwarzenthal, Moffitt, et al., 2021), ethnic–racial identity (Camacho et al., 2018; Del Toro & Wang, 2021), academic outcomes (e.g., achievement, motivation, Del Toro & Wang, 2021; Griffin et al., 2017), youth anti-racism actions (Bañales, Aldana et al., 2021), and socioemotional adjustment (e.g., sense of belonging, life satisfaction, Byrd, 2017; Celeste et al., 2019), among others.

To date, however, the different dimensions of cultural diversity climate (e.g., multiculturalism climate, colorblind climate, polyculturalism climate) have often been studied separately, preventing a complete understanding of their nature, and their connections and differences. In addition, cultural diversity climate and related concepts have been investigated in an array of disciplines (e.g., social, developmental, educational, cultural, work, and organizational psychology). However, despite the promise of interdisciplinary exchanges, some of these respective lines of research have developed in relative isolation from each other. Further, cultural diversity climate dimensions are complex and nuanced and can include different facets that capture distinct aspects of a cultural diversity climate dimension. However, at times, prior research has glossed over these nuances by not paying sufficient attention to the facet level of cultural diversity climate dimensions. This lack of attending to facets of diversity climate dimensions seems like a missed opportunity to amass more differentiated knowledge.

In this review, we aim to address these limitations. First, we distinguish cultural diversity climate from other diversity concepts to move toward greater conceptual clarity. We then provide an overview and discussion of the five core dimensions of cultural diversity climate (the cultural diversity climate “Big Five”: intergroup contact theory’s optimal contact conditions, multiculturalism climate, colorblind climate, critical consciousness climate, and polyculturalism climate) and their facets. For cultural diversity climate dimensions for which facets have not yet been introduced, we propose facets in order to prompt future empirical studies to test these extensions directly. We also identify similarities and differences between the cultural diversity climate dimensions and facets, and suggest an overarching metadimension matrix in which these dimensions and facets can be located. Next, we present different measurement approaches, including their strengths and weaknesses. The review concludes with a 10-point plan that offers suggestions for future research. We also highlight how the proposed integrative, multidimensional, and multifaceted framework can advance psychological theory on diversity climate.

## Positionality Statement

As authors, we acknowledge that our perspectives on the topic of cultural diversity climate are shaped by our backgrounds as White females from Central Europe, currently in our first faculty positions at a university. While our research is driven by a commitment to promoting equity and inclusion in educational settings, we recognize that our backgrounds and experiences within a predominantly White, European academic context may limit our ability to fully grasp the lived experiences of those from marginalized communities. We understand that our views may differ from those of individuals from other racial, ethnic, or geographic backgrounds. To address potential biases, we actively engaged with a diverse body of literature on cultural diversity, including perspectives from authors of underrepresented and marginalized groups, to inform our understanding and the content of this article. We have also sought feedback and engaged in discussions with colleagues from diverse backgrounds, which has helped us challenge our assumptions and critically reflect on our perspectives and how they are inevitably influenced by our social and cultural identities.

## Achieving Conceptual Clarity: Distinguishing Cultural Diversity Climate from Related Diversity Concepts

Cultural diversity climate describes how diversity is addressed in social settings and is manifested in the practices and messages (e.g., teaching practices aiming to encourage students to learn about other students’ cultures), policies (e.g., rules and principles adopted in a school, e.g., whether students are allowed to speak a language other than the national language), norms (e.g., general expectations of students, teachers, and other school staff about the ways cultural diversity is managed at school), and the general atmosphere (e.g., the “tone” and climate pervading a school) of a social setting (e.g., Abacioglu et al., 2019; Bardach et al., 2020; Byrd, 2017; Phalet & Baysu, 2020; Schachner, 2019; Verkuyten & Thijs, 2013). A defining aspect of cultural diversity climate is thus its focus on “the context” (e.g., the school setting) as opposed to the individual. This distinction is especially important because diversity climate measures still sometimes conflate contextual characteristics with personal features (e.g., one’s own beliefs about diversity, one’s own experiences with discrimination in a school), even though, according to the definition provided above, features of the individual should not be part of diversity climate measures (Baysu et al., 2016; Byrd, 2017). The conceptual distinction between cultural diversity climate and features of the individual also has methodological implications. Because cultural diversity climate refers to characteristics of the context (e.g., the school or class), it is conceptually meaningful to examine it at the context level (e.g., the school or class level) using appropriate statistical approaches such as multilevel models (e.g., Marsh et al., 2012). Nonetheless, studies on cultural diversity climate often use individual student ratings of cultural diversity climate without disentangling different levels of analysis (individual student and school/class level).

In the literature, many terms are related to cultural diversity climate (see Table 1). Table 1 shows that elements of the definition of cultural diversity climate in schools provided above (e.g., policy, norms) are also used by scholars from different disciplines (e.g., work and organizational psychology, social psychology) to refer to phenomena similar to a school’s cultural diversity climate but in contexts other than school settings (e.g., work units, national policy level). Like cultural diversity climate, these phenomena (diversity policies, diversity norms) all refer to the context. For example, *diversity policies* refer to nation-level policies that were developed to manage intergroup relations in societies (e.g., multiculturalism policy, colorblind policy; Guimond et al., 2013). The diversity policy term has also been used in work and organizational psychology research. For instance, diversity initiatives include a set of diversity policies aimed at improving the experiences and outcomes of target minority groups in the workplace (Leslie & Flynn, 2024). *Diversity norms* capture general expectations about the level of support that different diversity ideologies receive, which can differ from personally endorsed diversity beliefs/ideologies (Guimond et al., 2013), and they often focus on country-level diversity norms.

**Table 1.** Cultural Diversity Climate and Related Diversity Concepts: Definitions and Focus (Individual vs. Context).

Diversity concept	Definition	Focus
Diversity climate	Diversity climate = Approaches for addressing diversity, reflected in the practices, policies, norms, and the general atmosphere in social settings	Context
Diversity policy	Diversity policy = Nation-level policies that were developed to manage intergroup relations in societies; Guimond et al., 2013)	Context
Diversity norm	Diversity norm = General expectations about the level of support that different diversity ideologies receive	Context
Diversity cognitions/beliefs/ideologies	Diversity ideologies have been defined as distinct sets of beliefs capturing basic responses to diversity (Guimond et al., 2013; see also Rosenthal & Levy, 2010) or as people’s beliefs and practices regarding diversity (Rattan & Ambady, 2013; also referred to as diversity models). Leslie and Flynn (2024) defined diversity cognitions as beliefs about how to approach diversity and its effects. They further distinguished between diversity beliefs and ideologies as distinct types of individual diversity cognitions: - Diversity ideologies = Personal beliefs about how to approach diversity - Diversity beliefs = Personal beliefs about how diversity is beneficial; hence, diversity beliefs focus on the effects of diversity only	Individual
Diversity attitudes	Diversity attitudes = General positive or negative orientation toward diversity (De Meuse & Hostager, 2001; Leslie & Flynn, 2024; Nakui et al., 2011)	Individual
Diversity mindsets	Diversity mindsets = Multidimensional construct capturing knowledge of (a) a group’s diversity, (b) how group diversity could affect group processes and group performance, and (c) how one should approach this diversity (Homan, 2019; Van Knippenberg et al., 2013)	Individual
Acculturation strategies	Acculturation strategies = Strategies individuals use to deal with the acculturation process (Berry, 1997, 2007; see Kunst & Mesoudi, 2025, for recent extensions)	Individual

*Note.* Leslie and Flynn (2024) further considered diversity climate as a third type of diversity cognition that focuses on the context [here: work units] instead of the individual and is thus aligned with our definition of diversity climate (see also Holmes et al., 2021).

By contrast, other diversity concepts (e.g., diversity ideologies, diversity attitudes, see Table 1) clearly differ from cultural diversity climate in terms of the referent (i.e., they focus on individuals and thus, personal beliefs and experiences, rather than on contextual characteristics). *Diversity ideologies* have often been defined as sets of beliefs capturing basic responses to diversity (Guimond et al., 2013; see also Rosenthal & Levy, 2010). Nonetheless, diversity ideologies are still conceptually close to cultural diversity climate, as in some instances, a specific cultural diversity climate dimension can be considered a contextual representation of a cultural diversity ideology. For example, the statement “I think that racial, ethnic, and cultural group memberships do not matter very much to who we are” captures colorblindness as a personal ideology, whereas the statement “In this school, we are told that racial and ethnic group memberships do not matter very much to who we are” measures colorblind climate. In the next section, when we introduce the cultural diversity climate “Big Five,” we also cover conceptual links between personal diversity ideologies and specific diversity climate dimensions in more detail. For some dimensions, the link to a corresponding personal ideology or belief is more apparent than for others. This may be due, in part, to the fact that the development of the ideology construct and related theories preceded that of the corresponding climate construct (e.g., polyculturalism and polyculturalism climate). It should also be noted that diversity beliefs and ideologies have been conceptualized as distinct types of the overarching category of individual diversity cognitions (Leslie & Fynn, 2024; see Table 1 for details).

Further diversity concepts tied to the individual and not the context, include *diversity attitudes*, defined as general positive or negative orientation toward diversity, which including affective (e.g., liking diversity), behavioral (e.g., an individual’s tendency to seek out diversity), and cognitive (e.g., beliefs about diversity) components (De Meuse & Hostager, 2001; Leslie & Flynn, 2024; Nakui et al., 2011). Next, *diversity mindsets* capture individual knowledge on group diversity on multiple dimensions (e.g., knowledge of a group’s diversity and knowledge on how one should best approach this diversity, see Table 1). Finally, a further family of diversity concepts centering on individual experiences stems from research on acculturation strategies (e.g., cultural preservation, engaging with a new society, Berry, 1997, 2007; see Kunst & Mesoudi, 2025, for recent extensions).

## The Cultural Diversity Climate “Big Five”: Dimensions and Facets

The cultural diversity climate “Big Five” described herein bring together five diversity climate dimensions, similar to the Big Five framework of personality traits with its five dimensions and their facets (e.g., John & Srivastava, 1999; John et al., 2008). These five dimensions build on and expand theory and prior research on cultural diversity climate at school (e.g., Bañales, Lozada, et al., 2021; Byrd, 2017; Celeste et al., 2019; Schachner, Schwarzenthal, Moffitt, et al., 2021; Schwarzenthal et al., 2023), and related psychological literature (e.g., Plaut et al., 2018; Rosenthal & Levy, 2010; Pettigrew & Tropp, 2006):

I. Intergroup contact theory’s optimal contact conditions (equality, intergroup cooperation, positive contact).
II. Multiculturalism climate (acknowledging and valuing diversity, learning about one’s own, and other cultures).
III. Colorblind climate (disregarding diversity and ethnic, racial, and cultural group memberships, emphasizing similarities rather than differences, assimilation).
IV. Critical consciousness climate (fostering critical reflection, motivation, and action to challenge racism and inequities).
V. Polyculturalism climate (focusing on the dynamic nature of “culture,” and mutual influences between cultures).

Figure 1 provides an overview of the five dimensions (middle layer) and their facets (outer layer). The facets subsumed under optimal contact conditions, multiculturalism climate, and colorblind climate (except for power-evasion climate) have already been examined in the literature on cultural diversity climate. However, no facets have yet been introduced for critical consciousness climate and polyculturalism climate. An important theoretical contribution of our work is, therefore, to propose facets for critical consciousness climate and polyculturalism climate, and to suggest adding power-evasion climate (Neville et al., 2000) as a new facet to colorblind climate.

**Figure 1.**
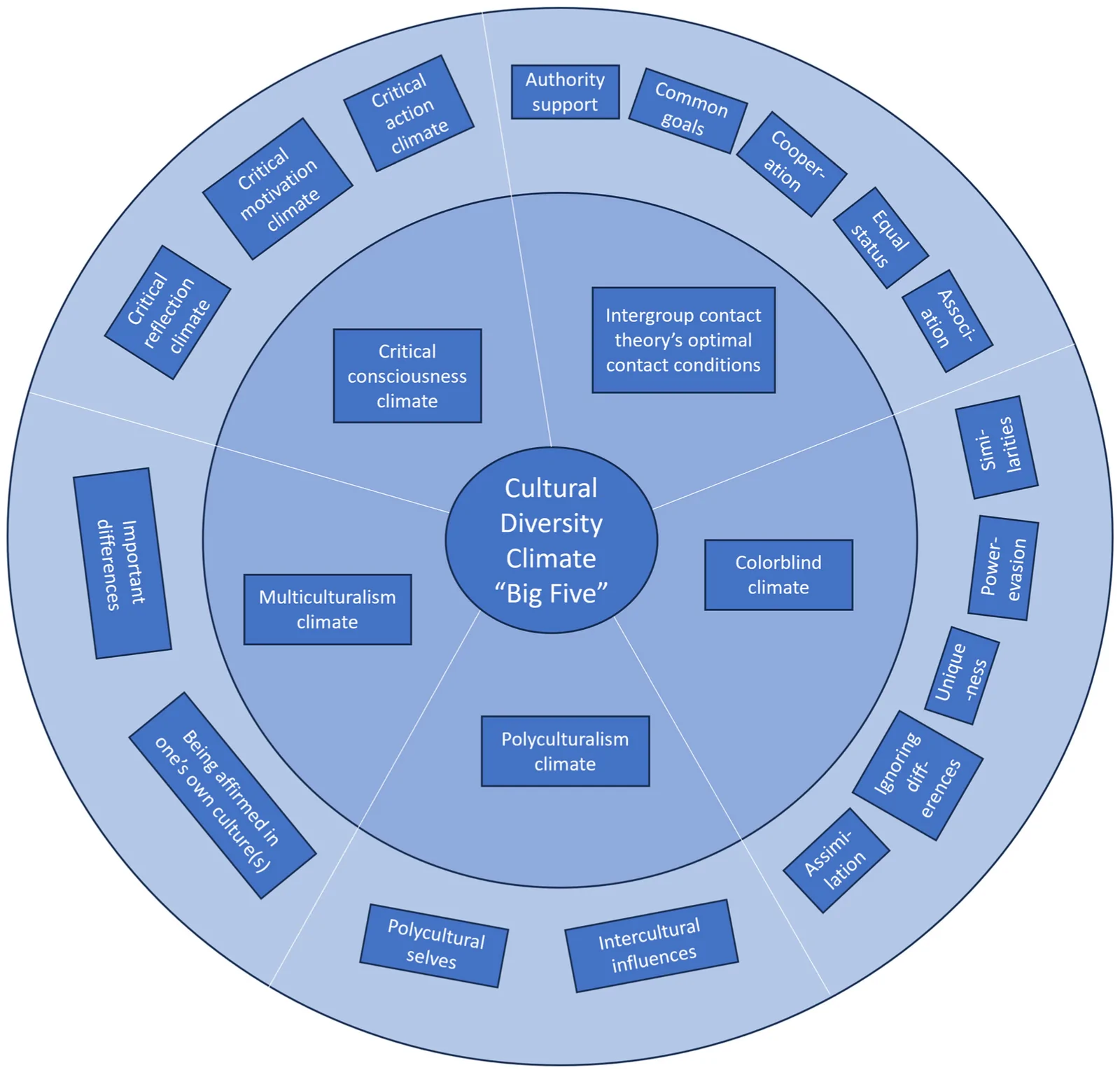
The cultural diversity climate “Big Five” dimensions (middle layer) and facets (outer layer).

Our primary aim in proposing this framework is to provide a coherent conceptual structure that organizes the key dimensions and facets of cultural diversity climate in school settings. At the same time, we see the framework as a foundation for theorizing the mechanisms, pathways, and potential barriers that influence how diversity climate elements emerge, as well as the implications they carry for students’ experiences and outcomes. These two aims—conceptual organization and theoretical explanation—are not mutually exclusive but mutually reinforcing. By articulating the dimensions and their facets, the framework enables a more holistic yet differentiated exploration of how specific aspects of climate may interact, align, or compensate for one another.

Overall, we largely take inspiration from research on the Big Five personality traits for our conceptualization of the cultural diversity climate “Big Five,” their structure (dimensions and, crucially, facets), and for discussing measurement issues (e.g., interstitial facets). Yet, we want to draw attention to key differences between cultural diversity climate dimensions and the Big Five personality traits, as diversity climate pertains to contextual factors, while personality traits refer to individual-level characteristics. Thus, we acknowledge that diversity climate dimensions may be considered to be conceptually closer to research on the psychological characteristics of situations (and not persons) within personality psychology (e.g., Bem & Funder, 1978; see Rauthmann et al., 2014). For instance, the DIAMONDS taxonomy (Duty, Intellect, Adversity, Mating, pOsitivity, Negativity, Deception, and Sociality) outlines eight dimensions on which people perceive, describe, and evaluate situations (Rauthmann et al., 2014). Similarly, diversity climate dimensions may be understood as situation (or context) characteristics pertaining to aspects of ethnic, racial, and cultural diversity.^1^

### Intergroup Contact Theory’s Optimal Contact Conditions: Fostering Positive Intergroup Contact

Intergroup contact theory, a prominent social-psychological theory, has inspired a great amount of research on cultural diversity climate in schools. According to intergroup contact theory, positive intergroup contact between members from different groups is effective in fostering positive intergroup outcomes (e.g., reduced prejudice, Allport, 1954; Pettigrew & Tropp, 2006; Tropp et al., 2017). Further, a set of *optimal contact conditions* have been proposed. These contact conditions specify the circumstances under which intergroup contact is especially likely to unfold its beneficial effects (Allport, 1954; Pettigrew & Tropp, 2006; Tropp et al., 2022), even though clear insights into their relative effectiveness based on experimental evidence are still lacking (see Paluck et al., 2019, for a meta-analysis of high-quality contact studies featuring random assignment and delayed outcome measures).

The *equal status condition* holds if members from different groups have equal status at school and are equally respected. In school contexts, this condition could, for example, be reflected in teachers’ equal and respectful treatment of all students, irrespective of their background. By contrast, situations in which teachers treat students from minority group less favorably indicate the absence of equal status. The *common goals condition* requires students from different groups to work together to achieve shared goals. The *cooperation condition* emphasizes the importance of collaboration and exchange among different groups. Interdependent learning strategies that students with different backgrounds use to work toward jointly developed learning goals are an example of how both the common goals and cooperation conditions can be implemented in school settings (Tropp et al., 2022). Support for intergroup contact from authority figures (e.g., teachers or principals, *authority support condition*) shows students which behaviors are deemed appropriate (Pettigrew, 1998). For instance, a teacher establishing classroom rules that ensure that students from all different backgrounds have the opportunity to participate fully in classroom activities serves as an indicator of authority support (which is, in this case, closely linked to the equal status condition). The *association* condition (Pettigrew, 1998), applied to school settings, specifies that at school, individuals from different groups should have the chance to form friendships. A school in which largely segregated friendship groups are considered the norm or are even emphasized, violates the association condition. The different contact conditions (which we conceptualize as facets) can sometimes be difficult to disentangle in applied settings and are often conceptualized as interrelated and mutually reinforcing (Pettigrew & Tropp, 2006; Tropp et al., 2022).

### Multiculturalism Climate: Learning About Different Cultures and Being Affirmed in One’s Own Culture(s)

In a school in which a *multiculturalism climate* pervades, cultural variation is valued, and children learn that concepts such as culture, race, or ethnicity represent important aspects of people’s lives (Rosenthal & Levy, 2010; Schachner et al., 2016). Although multiculturalism climate as a cultural diversity climate dimension focuses on the (school) context, multiculturalism climate still shares conceptual ties with the personal diversity ideology of multiculturalism, that is, personal beliefs that group differences are to be acknowledged and celebrated (Guimond et al., 2013; Rosenthal & Levy, 2010; Sasaki & Vorauer, 2013; Whitley & Webster, 2019). Hence, taking a closer look at personal multiculturalism ideologies is also useful for better understanding multiculturalism climate and its facets. First, multiculturalism as a personal ideology can entail the importance individuals attach to learning about and drawing attention to cultural differences, thereby enhancing their understanding of the lives and perspectives of diverse others (important differences; Rosenthal & Levy, 2010). Multiculturalism as a personal ideology can also center on learning to value the positive contributions of different groups to a diverse society (appreciating contributions; Rosenthal & Levy, 2010). Lastly, multiculturalism as a personal ideology can be about groups (e.g., immigrants in a new country) maintaining their own cultures (maintaining cultures; Berry & Kalin, 1995; Rosenthal & Levy, 2010; Ryan et al., 2010; Wolsko et al., 2006).

Mirroring multiculturalism as a personal ideology with a focus on the importance of differences on a contextual level, multiculturalism climate at school often involves teaching about cultural variations and encouraging students to learn about different cultures, customs, and traditions (referred to here as the *important differences facet* of multiculturalism climate, Rosenthal & Levy, 2010; see also Bardach et al., 2024). A second type of multiculturalism climate that can be found in schools specifically focuses on providing opportunities for (minority) children and adolescents to learn about, be affirmed in, and value their own culture(s); here, we label this facet of multiculturalism climate as the *being affirmed in one’s own culture(s) facet*. This facet of multiculturalism climate can be realized by integrating examples from students’ cultures into the curriculum and supporting students to engage with and learn about the history of their own culture(s). As the being affirmed in one’s own culture(s) facet helps ethnic, racial, and cultural minority students maintain their own cultures and traditions, it shares connections with multiculturalism climate as a personal ideology with a focus on maintaining cultures. At the same time, the being affirmed in one’s own culture facet of multiculturalism climate is conceptually close to the personal multiculturalism ideology with a focus on appreciating contributions: In a school or classroom in which attention is devoted to integrating elements of ethnic, racial, and cultural minority cultures, all students are prompted to value and appreciate how different cultural groups have contributed and continue to contribute to society (Rosenthal & Levy, 2010; Ryan et al., 2007; Wolsko et al., 2006).

Additionally, the being affirmed in one’s culture facet can be linked to the literature on parental ethnic–racial socialization (e.g., Byrd, 2017; Saleem & Byrd, 2021), which further deepens the comprehension of this multiculturalism climate facet. Parental ethnic–racial socialization describes a collection of practices parents use to communicate information, beliefs, and values about ethnicity, culture, and race to their children. Parental ethnic–racial socialization has primarily been studied in the United States, with a focus on parents of color (Hughes et al., 2007; Huguley et al., 2019; Lesane-Brown, 2006), even though there has also been an increased interest in ethnic–racial socialization practices of white parents in recent years (e.g., Loyd et al., 2018; Nieri et al., 2024). One of the most commonly endorsed types of parental ethnic–racial socialization is pride and heritage socialization (also referred to as cultural socialization), which resembles the being affirmed in one’s own culture(s) facet of multiculturalism. Pride and heritage socialization refers to parenting approaches that proactively promote cultural pride and knowledge by teaching children about their heritage, cultural customs, and history (Hughes et al., 2007; Huguley et al., 2019), thus nurturing and affirming ethnic–racial identities that can buffer the adverse impact of discrimination.

### Colorblind Climate: Disregarding Cultural Diversity

In a *colorblind climate*, diversity is disregarded. Colorblind climate is the most heterogeneous of all cultural diversity climate dimensions on the facet level. Again, to help readers grasp what the respective colorblind climate facets are, it is helpful to refer to the literature on personal diversity ideologies that parallel the colorblind climate facets. First, in their overview of diversity ideologies, Rosenthal and Levy (2010) outlined that “in its most generic form, colorblindness suggests ignoring or avoiding discussion of group categories” (p. 218). Similarly, one facet of colorblind climate centers on interactions at school and teaching practices that elide discussions of group categories and cultural diversity (*ignoring differences facet*, e.g., Civitillo et al., 2021).

Second, colorblindness as a personal diversity ideology can also take the form of emphasizing similarities among groups of people and a common ingroup identity (e.g., “we are all members of a specific nationality”; see also early research on the Common Ingroup Model, Gaertner & Dovidio, 2000; Rosenthal & Levy, 2010). The related cultural diversity climate facet (*similarities facet*) includes teaching practices and messages emphasizing that similarities between students are more important than cultural differences or that, for example, “we are all people, irrespective of where we are from” (Schachner, Schwarzenthal, Moffitt, et al., 2021; see also Albarello et al., 2021, on the concept of developing identification with humanity).

A third type of colorblind ideology and related facet of colorblind climate advocates that all groups should adopt the dominant culture (i.e., assimilation, Rosenthal & Levy, 2010). In a school in which the *assimilation facet* of colorblind climate prevails, students might not be allowed to speak a language other than the national language (Baysu et al., 2016). For some scholars, colorblindness is multifaceted and encompasses assimilation (Gündemir et al., 2017; Rosenthal & Levy, 2010), whereas others consider colorblindness (often focusing on simply ignoring group categories, i.e., the ignoring differences facet, or focusing on commonalities, i.e., the similarities facet) and assimilation to be distinct (Hahn et al., 2015). We take the first stance here as both disregard cultural diversity, although they take different directions, with colorblind climate in the assimilation facet trying to push everyone toward the majority culture and colorblind climate in the ignoring differences and similarities facets more broadly dismissing diversity and cultural differences. We also acknowledge the connections between these different colorblind climate facets. For example, by ignoring differences and emphasizing similarities, the majority culture still remains the implicit, unquestioned norm, which implies assimilation (e.g., Dovidio et al., 2016; Neville et al., 2013).

Fourth, colorblindness—both as a personal ideology and as a colorblind climate facet—can center on individual differences (as opposed to group categories). This facet of colorblind climate (termed *uniqueness facet*) manifests, for example, in a school climate and teacher practices that communicate to students that each person is unique (Byrd, 2015; Celeste et al., 2019). However, research on the uniqueness facet of colorblind climate in schools is rare. Neville et al. (2013) integrated the ignoring differences, similarities, and uniqueness elements of colorblindness into the concept of color-evasion as a personal colorblindness ideology. In a similar vein, in school-based research, the ignoring differences and similarities facets have been merged to form a color-evasion climate measure (Juang et al., 2020; Schachner, Schwarzenthal, Moffitt, et al., 2021).

Fifth, colorblindness as a personal ideology can also come in the form of power-evasion, in which the existence of institutional racism is denied, minimized, and distorted (Neville et al., 2000, 2013; Yi et al., 2023). Power-evasion has not yet been systematically integrated into the colorblind climate measures that have been developed for school settings. Nonetheless, it is important to acknowledge power-evasion, as the *power-evasion facet* of colorblind climate would go beyond ignoring culture, race, and ethnicity (see the ignoring differences facet) by including teacher practices and a school climate in which (institutional) racism and structural inequities are denied. For instance, in a school characterized by the power-evasion facet of colorblind climate, teachers could negate, downplay, and misrepresent blatant forms of racism (e.g., by emphasizing that racism may have been a problem in the past but is no longer a problem today), institutional racism (e.g., by claiming that ethnic, racial, and cultural minorities unfairly benefit from certain policies and practices, and creating an atmosphere at school in which such views are spread and reinforced), and racial privilege (e.g., by telling students that members from ethnic, racial, and cultural majority groups do not have certain advantages, Neville et al., 2000). Just like power-evasion as a personal ideology (Yi et al., 2023), the power-evasion facet of colorblind climate seems likely to breed intolerance, prejudice, and racism among students from ethnic, racial, and cultural majority groups and to increase internalized oppression among minority students. Nonetheless, studying power-evasion at the context-level (i.e., as a colorblind climate facet) will provide unique and critical information that cannot be captured by assessing power-evasion as a personal ideology. For example, a student with an ethnic, racial, and cultural minority background might not endorse power-evasion as a personal ideology themselves but may perceive a strong power-evasion climate at their school.

### Critical Consciousness Climate: Reflecting on and Challenging Racism and Inequities

In a critical consciousness climate, social inequity should be actively discussed and reflected upon (Schwarzenthal et al., 2022). Hence, schools characterized by a critical consciousness climate teach students and encourage discussions about systemic inequities, racism, and other forms of discrimination and how to redress them (Bañales, Lozada, et al., 2021; Byrd, 2017; Schachner, Schwarzenthal, Moffitt, et al., 2021). The concept of critical consciousness climate is derived from work by Freire (1973, 2000) and builds on research on critical consciousness as a personal-level construct. Critical consciousness describes how individuals, especially oppressed or marginalized people, learn to critically analyze their social conditions and act to change them (Freire, 1973, 2000; see also Diemer et al., 2016; Diemer & Rapa, 2016; Heberle, Rapa, & Farago, 2020; Watts et al., 2011). Critical consciousness as a person-level construct includes three aspects, namely, critical reflection (questioning social arrangements and structures that uphold marginalization), critical motivation (feeling motivated and perceiving oneself as capable of effecting social change), and critical action (individually or collectively actively engaging to change social inequities). Critical action is often assessed as intended action, as school students may face barriers to engaging in certain forms of critical action available to adults (e.g., voting, Freire, 2000; Heberle, Rapa, & Farago, 2020; Jemal, 2017; Schwarzenthal et al., 2022).

So far, critical consciousness climate at school has primarily been operationalized as a unidimensional climate dimension that strongly focuses on the critical reflection component (here referred to as the *critical reflection facet*, e.g., “Teachers teach about social inequality in the United States,” Byrd, 2017; “In class we talk about how people from different backgrounds do not always have the same opportunities in Germany,” Schachner, Schwarzenthal, Moffitt, et al., 2021). However, to broaden the scope of research on critical consciousness climate, it may be worthwhile to consider additional critical consciousness climate facets that are aligned with the respective personal critical consciousness aspects of critical action and critical motivation. Relevant practices have been identified by qualitative studies (e.g., in-depth case studies of schools, Seider & Graves, 2020) and could feed into theoretical accounts of critical consciousness climate and the respective scales that can be administered in quantitative investigations.

Specifically, the *critical action facet* of a school’s critical consciousness climate could foreground whether discussions of actual (or intended) critical actions (e.g., participating in or planning a public event or a demonstration for a political or social cause, Eckstein et al., 2015) take place at school. Also, some schools may offer students hands-on, guided experiences with political engagement and activism (e.g., writing letters to state representatives to address issues of systemic inequity and racism, project-based classes aimed at changing practices/policies within a certain school). Furthermore, teachers who are politically active can become important role models who might inspire students to engage in critical actions themselves (Seider & Graves, 2020). However, we caution that teachers should be mindful not to push students toward a specific political direction and should address controversial societal issues in a way that reflects their inherent complexity, while still fulfilling their duty to protect human rights and speak out against discrimination and racism.

The *critical motivation facet* of a school’s critical consciousness climate could center on educational practices that foreground and strengthen students’ motivations and beliefs that they are capable of navigating, challenging, and resisting oppressive systems (see also research on motivational climate without an explicit focus on “culture,” Bardach et al., 2020). For instance, teachers could communicate to students that their opinions are important and that they can make a difference, for example, by addressing problems in their communities, and, ultimately, by contributing to changing unjust societies. Also, creating safe spaces (e.g., a school theater play, Seider & Graves, 2020) allows students to practice how to challenge social inequities and oppressive systems and fosters respective motivations. Of course, in real-life school settings, the three facets of critical consciousness climate may overlap and are likely to mutually reinforce each other (e.g., motivationally supportive practices may lead to or blend into critical action practices).

### Polyculturalism Climate: Emphasizing Connections Between Cultures

A *polyculturalism climate* promotes awareness of cross-cultural influences and the dynamic, changing nature of cultures (Rosenthal et al., 2019). When a polyculturalism climate is present, discussions about how individuals can be influenced by, and belong to, more than one culture are encouraged at school, and students learn about past and current interactions and connections among cultures and groups (Rosenthal & Levy, 2010; Schachner, Schwarzenthal, Moffitt, et al., 2021). The concept of polyculturalism climate stems from research on polyculturalism as a personal diversity ideology (Kelly, 1999; Prashad, 2003; Rosenthal & Levy, 2010) that captures the personal belief that cultures are the products of past and current interactions that take place between different groups and that, due to intersecting histories, everyone is connected to others from other cultures. Whereas more research exists on polyculturalism as a personal ideology (e.g., Bernardo & Presbitero, 2017; Cho et al., 2017; Rosenthal & Levy, 2010), polyculturalism climate at school has largely remained unexplored so far (for notable exceptions, see Juang et al., 2020; Schachner, Schwarzenthal, Moffitt, et al., 2021).

To date, both polyculturalism climate and polyculturalism as a personal ideology have been measured as unidimensional constructs. Here, we suggest the possibility of bifurcating the polyculturalism climate construct into two distinct yet related facets (with empirical tests of this distinction lying ahead). The *intercultural influences facet* could include discussions, messages, and practices at school emphasizing that cultures have always influenced each other and continue to do so, and that this can be valuable. Learning and focusing on the historical and continued connections between different racial, ethnic, and cultural groups at school can improve students’ understanding of history and the present, and also has the potential to improve intergroup attitudes and relations (Rosenthal & Levy, 2010). Whereas the intercultural influences facet focuses on the level of cultures, another facet, which we label the *polycultural selves facet*, focuses on the individual (Morris et al., 2015). The polycultural selves facet involves discussions, messages, and practices at school that recognize individuals as culturally complex, and their cultural identities as dynamic and malleable. In a school or class characterized by the polycultural selves facet of polyculturalism climate, it is acknowledged that people have partial and plural engagements with different cultures and thus, take influences from multiple cultures (Morris et al., 2015, see also conceptualizations of multiple cultural identities, Hong & Zhan, 2019; Kang & Bodenhausen, 2015; van Oudenhoven & Benet-Martinez, 2015). The polycultural selves facets directs students’ attention to how individuals, including themselves, pick up proficiencies from multiple cultures throughout their life through a range of different learning processes (Morris et al., 2015).

Table 2 presents sample items for the five cultural diversity climate dimensions and their facets.

**Table 2.** Five Cultural Diversity Climate Dimensions and Their Facets: Sample Items from Existing Measures.

Cultural diversity climate dimension and facets	Sample items
Intergroup contact theory’s optimal contact conditions	
Authority support facet: “In this class the teacher encourages children to make friends with children from other countries” (Jugert et al., 2011)	
Equal status facet: “In this class, the teacher is fair to students of all races” (Molina & Wittig, 2006)	
Cooperation facet: “Students of different races/ethnicities study together” (Byrd, 2017)	
Common goals facet: “In this class children from different countries all work together for the same things” (Jugert et al., 2011)	
Association facet: “In this class, students of different races just do not like being together” (reverse coded; Molina & Wittig, 2006)	
Multiculturalism climate	
Important differences facet: “At your school, they encourage you to learn about different cultures” (Byrd, 2017)	
Being affirmed in one’s own culture facet: “My teacher(s) use examples from my culture when teaching” (Chun & Dickson, 2011)	
Colorblind climate	
Ignoring differences facet: “At your school, people think race/ethnicity is not an important factor in how people are treated” (Byrd, 2017)	
Assimilation facet: “In my school speaking another language than Dutch is not tolerated” (Baysu et al., 2021)	
Similarities facet: “In school we are taught that we are all people, regardless of where we are from” (Schachner, Schwarzenthal, Moffitt, et al., 2021)	
Power-evasion facet: There are currently no items from studies in school contexts available	
Critical consciousness climate	
There are no scales available yet that capture separate facets of critical consciousness climate at school described in our manuscript (critical reflection facet, critical action facet, critical motivation facet). Existing items assessing critical consciousness climate (see below for examples) mainly tap into the facet we label as critical reflection facet and were therefore assigned to this facet	
Critical reflection facet: “In your classes you have learned how race/ethnicity play a role in who is successful (Byrd, 2017); “In school we talk about how people from certain heritage cultures have to work harder to get a good job” (Schachner, Schwarzenthal, Moffitt, et al., 2021)	
Critical action facet and critical motivation facet: No scales from school contexts available yet	
Polyculturalism climate	
Scales designed to separately capture the facets of polyculturalism climate at school described in our manuscript (intercultural influences, polycultural selves) are currently not available. We therefore assigned existing items to these two facets, even though more work would be needed to clearly align the items with the facets we proposed	
Intercultural influences: “In class we learn about how cultures in Europe and the rest of the world have influenced and continue to influence each other” (Schachner, Schwarzenthal, Moffitt, et al., 2021)	
Polycultural selves: “In school we talk about how people can be influenced by more than one culture” (Schachner, Schwarzenthal, Moffitt, et al., 2021)	

*Note.* Although the exact response options can vary between studies, scales assessing cultural diversity climate dimensions and facets typically adopt a Likert-scale format (e.g., ranging from 1 = *strongly disagree* to 5 = *strongly agree*).

## Differences and Connections Between Cultural Diversity Climate Dimensions and Facets

Some cultural diversity climate dimensions and facets consist of contrasting elements.

For example, at the level of *dimensions*, both multiculturalism climate and critical consciousness climate can be conceptualized as opposing poles of colorblind climate. When considering different colorblind climate *facets*, we argue that multiculturalism climate may best be conceptualized as the opposite of the colorblind climate facets ignoring differences, whereas critical consciousness climate may best be conceptualized as the opposite of the herein newly introduced colorblind climate facet power-evasion.

On the other hand, there is also some overlap between specific dimensions and facets. At the level of *dimensions*, colorblind climate can be conceptually linked to intergroup contact theory’s optimal contact conditions, as the promotion of positive contact, cooperation, and ensuring equality (i.e., optimal contact conditions) can coincide with deliberately neglecting cultural variations (i.e., colorblind climate; Pettigrew, 1998; Schachner et al., 2016; Schofield, 2010). Similarly, (early) research on the Common Ingroup Model (Gaertner et al., 1994; Gaertner & Dovidio, 2000) suggested that establishing optimal contact conditions helps transform group members’ perceptions of group boundaries (i.e., moving from “us” and “them” to a more inclusive “we,” Gaertner et al., 1994). Moving to the *facet* level of colorblind climate, we argue that the situation described by Gaertner, Dovidio, and colleagues is particularly well-aligned with the similarities facet of colorblind climate. Hence, equal status and other contact conditions do not have to imply “sameness” (see e.g., Tropp et al., 2021 showing how contact can be complemented with discussions of group-based hierarchies), but they can. We propose that certain facets (e.g., equal status from optimal contact conditions, the similarities facet from colorblind climate) may even function as interstitial facets (see Bainbridge et al., 2022 in the context of the Big Five personality traits), that is, facets that share variance with more than one cultural diversity climate dimension. Nonetheless, despite some overlap between (not well-implemented) contact conditions and colorblind climate, we consider intergroup contact theory’s optimal contact conditions to be an overall positive cultural diversity climate dimension, whereas colorblind climate—and especially its assimilation, power-evasion, and ignoring differences facets—is not.

Some dimensions or facets of diversity climate compensate for limitations inherent in other dimensions or facets. At the level of diversity climate *dimensions*, multiculturalism climate can be considered a cultural diversity dimension that addresses important blind spots of the optimal contact conditions dimension. Specifically, the climate in schools should be about not only equality and positive contact but also explicitly acknowledging and valuing cultural variations and engaging with one’s own cultural background (Byrd, 2017; Rosenthal & Levy, 2010). Considering multiculturalism climate on the *facet* level further allows researchers to drill deeper into the meaning of this diversity climate dimension. The multiculturalism facet of learning about other cultures is important for all students to broaden their horizons in ways that optimal contact conditions likely cannot achieve. By contrast, the being affirmed in one’s own culture facet can become a particularly important resource for children and adolescents from ethnic, racial, and cultural minority groups, as their identities and backgrounds are valued in the school setting. In addition, when we take a step back to focus on the level of *dimensions* again, it becomes clear that although polyculturalism climate and multiculturalism climate share some overlap, polyculturalism climate can overcome specific limitations of multiculturalism climate. For example, multiculturalism climate may suggest that cultures are fixed and categorical, whereas polyculturalism climate emphasizes the fluidity of culture(s). A multiculturalism climate can, at times, reinforce stereotypes by presenting cultures as bounded groups defined by distinct, shared, and stable features (e.g., important differences facet). In contrast, a polyculturalism climate challenges this essentialist view by emphasizing the dynamic, interconnected nature of cultures and the ways they influence and shape one another through ongoing contact and exchange (Morris et al., 2015; Prashad, 2003; Rosenthal et al., 2019).

Further ideas on compensatory effects center on critical consciousness climate. In an ideal world, individuals should be treated fairly regardless of race, ethnicity, cultural background, and social position (see optimal contact conditions dimension); variations should be considered as an important resource (see multiculturalism climate dimension); and connections between cultures should be acknowledged (see polyculturalism climate dimension). Unfortunately, education systems in many parts of the world are far from achieving this ideal. Instead, they continue to support espoused ideologies, structures, and policies that contribute to individual and systemic discrimination and inequity (Castro-Atwater, 2020; Tessema et al., 2025). Accordingly, critical consciousness climate takes on a crucial role and provides a remedy for the shortcomings of the other dimensions. For instance, on the level of *dimensions*, optimal contact conditions lack explicit discussions of the social inequities that ethnic, racial, and cultural minority students may face, and the same can apply to multiculturalism climate, especially if it is only superficially implemented (see also, e.g., Bañales, Aldana, et al., 2021; Byrd, 2017; Schachner et al., 2021; Schwarzenthal et al., 2022). Scholars have also cautioned that favorable contact conditions and the resulting positive intergroup experiences may lead members of disadvantaged groups to downplay the extent of injustice and discrimination their group faces, thereby reducing their support for efforts to challenge inequity (Dixon et al., 2010).

Additional insights can be derived by focusing on the content of specific *facets*. For example, the multiculturalism climate facets emphasize aspects such as learning about and valuing other cultures and cultural differences (see the important differences facet). Now consider, in particular, the newly introduced critical consciousness facets (critical action and critical motivation), which add elements such as fostering students’ motivation to address inequities and discussing—or taking—concrete actions. Thus, the critical consciousness facets diverge from multiculturalism climate, not only in terms of content but also in their orientation toward motivational and action-oriented components.

Also, polyculturalism climate primarily emphasizes positive, mutually beneficial interactions between cultures, rather than negative influences such as those rooted in colonialism or the historical domination of certain groups by others. Therefore, we argue that it should be accompanied by a critical consciousness climate to address both historical and contemporary injustices. This may be necessary to avoid the risk that discussions of cultural influences within a polyculturalism climate (see especially the intercultural influences facet of polyculturalism climate) drift toward the notion that one culture is inferior to another (see the power evasion facet of colorblind climate) and should therefore blend into the dominant culture (see the assimilation facet of colorblind climate).

To conclude, while the diversity climate “Big Five” and their facets are conceptually distinct, they are interconnected in various ways, sometimes functioning as opposing poles and at other times aligning through shared conceptual elements or compensating for each other’s limitations. This reinforces the value of a multidimensional and multifaceted approach to diversity climate in capturing the complexity of how individuals perceive and experience diversity-related features of their environment.

## Theorizing on Diversity Climate Dimension and Facet Interconnectedness: The Role of Cultural Diversity Climate Metadimensions

As we take inspiration from personality psychology throughout the article, we also aim to briefly discuss the notion of dimension and facet interconnectedness against the background of developments in personality psychology research on its Big Five. The interconnectedness between diversity climate dimensions represents a notable departure from traditional personality frameworks, in which the Big Five were typically viewed as semi-independent, or, at least, not as causally dependent on one another (e.g., Saucier, 2002). Our understanding of diversity climate aligns more closely with conceptualizations of personality traits which recognize that traits are, to some extent, interconnected while still maintaining their distinctiveness. At this juncture, however, it is worth noting that there is research on the structure of personality that has attributed the pattern of correlations among the Big Five to two *metatraits* (i.e., higher-order factors above the Big Five): Alpha and Beta (Digman, 1997), or Stability and Plasticity (DeYoung, 2006), which include Openness and Extraversion (Plasticity) and Conscientiousness, Agreeableness, and Neuroticism (Stability).

Relatedly, how can theorizing on diversity climate *metadimensions* advance current understanding? For example, in previous work, two overarching dimensions have been proposed (e.g., Schachner et al., 2016; Schachner, Schwarzenthal, Moffitt, et al., 2021), although factor-analytical work that has empirically examined such metadimensions (e.g., by comparing different factor models) seems absent from the literature. The two meta-dimensions of Schachner et al. (2016), Schachner, Schwarzenthal, Moffitt, et al. (2021) were labelled as “equality and inclusion” (including optimal contact conditions and the similarities facet of colorblind climate) and “cultural pluralism” (including multiculturalism climate, the critical reflection facet of critical consciousness climate, and polyculturalism climate). These metadimensions are conceptually meaningful when considering the content of each dimension (or the facets used to represent a specific dimension, such as the similarities facet for colorblind climate).

Nonetheless, these previously introduced metadimensions may not yet fully capture the multidimensional and multifaceted conceptualization of diversity climate adopted in our work. Due to the focus on expanding the facet-level of diversity climate, the dimensions put forth in our work are more fine-grained than those of Schachner and colleagues: All dimensions in Schachner, Schwarzenthal, Moffitt, et al. (2021) are assessed with only one of the facets discussed here, except for contact conditions, whereas we include overall 17 facets for the five dimensions. Schachner, Schwarzenthal, Moffitt, et al. (2021) subsume contact conditions and the similarities facet of colorblind climate under the “equality and inclusion” metadimension. The other colorblind climate facets described herein (e.g., power-evasion, assimilation) are not included, and more difficult to reconcile with “equality and inclusion.” In addition, “cultural pluralism” includes multiculturalism climate, polyculturalism climate, and critical consciousness climate. However, we argue that critical consciousness climate and polyculturalism climate, especially if enriched with multiple additional facets to broaden the respective construct space, are “more” than cultural pluralism/multiculturalism climate. For example, while discussing social inequity is sometimes assumed to be part of cultural pluralism approaches, it is often not included in practice (e.g., Agirdag et al., 2016). Moreover, empirical studies show differential effects of valuing cultural diversity and fostering discussions of social inequity. For instance, Schwarzenthal et al. (2022) found that multiculturalism climate was largely unrelated to students’ personal critical consciousness once critical consciousness climate was taken into account.

Here, we propose an alternative way of conceptualizing metadimensions in diversity climate research. Specifically, diversity climate dimensions and their facets can be situated along multiple metadimensions simultaneously and dynamically, resulting in a *metadimension matrix*. Examples include the identity-blind versus identity-conscious metadimension (see also research on diversity ideologies, Leslie & Flynn et al., 2022). Multiculturalism climate, polyculturalism climate, and critical consciousness climate are identity-conscious, colorblind climate is identity-blind, and contact conditions may be placed somewhere in the middle of the continuum (see also Bardach et al., 2026). Another potential metadimension could focus on assimilation (see respective colorblind climate facet) versus inclusion (optimal contact conditions, multiculturalism climate, polyculturalism climate, similarities facet of colorblind climate) versus transformation (critical consciousness climate). It is also possible to conceptualize a metadimension focused on the self (e.g., being affirmed in one’s own culture) versus structural issues (e.g., critical consciousness climate facets, the power-evasion facet of colorblind climate, which taps into the *absence* of considering structural issues), or a metadimension contrasting the ignoring of inequity (colorblind climate) with the addressing of inequity (critical consciousness climate).

Table 3 provides a non-exhaustive overview of potential metadimensions. We argue that the focus on multiple metadimensions offers benefits for theory and research. Applying a multiple metadimension perspective allows us to account for the inherent complexity and interconnectedness of dimensions and facets. Depending on the metadimension and its underlying theoretical focus, specific dimensions and facets can be grouped together, and different grouping scenarios evolve depending on the metadimension one looks at. This also implies that, for example, the same two dimensions or facets may be grouped closely together on one metadimension but not on another metadimension. This can also explain why, sometimes, specific dimensions and facets go together, and sometimes they do not (e.g., multiculturalism climate and critical consciousness climate). Also, as each single dimension and facet can be located on multiple different metadimensions within the metadimension matrix, we can obtain a comprehensive picture of a dimension’s or facet’s characteristics. In addition, making the metadimensions explicit could also help in theorizing the relationships between cultural diversity climate and outcomes. For example, drawing on social identity theory (Tajfel & Turner, 1986; see also Phalet & Baysu, 2020), identity-conscious dimensions are likely to have different effects on students’ ethnic–racial identity development compared to identity-blind dimensions.

**Table 3.** Examples of Metadimensions Forming the Metadimension Matrix of Diversity Climate.

Metadimension	Diversity climate dimensions and facets
Identity-blind vs. identity-conscious	Identity-blind (colorblind climate) vs. identity-conscious (multiculturalism climate, polyculturalism climate, critical consciousness climate)
Cultural diversity minimizing vs. embracing	Cultural diversity minimizing (colorblind climate) vs. embracing (multiculturalism climate, and particularly its important differences facet)
Assimilation vs. inclusion vs. transformation	Assimilation (assimilation facet of colorblind climate) vs. inclusion (multiculturalism climate, polyculturalismus climate, contact conditions) vs. transformation (critical consciousness climate)
Essentialist vs. constructivist view of group membership	Essentialist (sometimes multiculturalismus climate) vs. constructivist (polyculturalism climate, critical consciousness climate) view of group memberships
Focus on social interactions among individuals vs. structural focus	Individual-level focus (contact conditions, colorblind climate facets except for power-evasion) vs. structural focus (critical consciousness, power-evasion facet of colorblind climate)
Alignment with diversity philosophies vs. concrete, observable practices	Alignment with diversity philosophies (multiculturalism climate, polyculturalism climate, colorblind climate) vs. concrete practices (contact conditions, critical action facet of critical consciousness climate)
Focus on the self vs. societal issues	Focus on the self (being affirmed in one’s own culture facet of multiculturalism climate, polycultural selves facet of polyculturalism climate) vs. societal issues (critical consciousness climate facets)

Lastly, when reflecting on the structure and interconnectedness of cultural diversity climate dimensions, a development in personality psychology and psychopathology should be highlighted that may have implications for theory and research on cultural diversity climate: the emergence of network approaches (e.g., Borsboom & Cramer, 2013; Christensen et al., 2020; Pe et al., 2018). For instance, in network approaches to psychopathology, disorders are a consequence of the causal interplay between symptoms (Borsboom & Cramer, 2013). Similarly, within a network approach, personality traits can be modeled as complex systems composed of many behavioral components that interact with one another (see, e.g., Christensen et al., 2020, for an empirical study). Thus, applying a network perspective may allow diversity climate dimensions to be conceptualized and studied as causal systems of mutually reinforcing classroom processes, teacher–student interactions, norms, and other dynamics within schools and classes, with blurred boundaries between dimensions (see also, for example, research on comorbidity in psychopathology; Cramer et al., 2010).

## Measuring Cultural Diversity Climate

How should a complex construct such as cultural diversity climate be assessed? We consider the discussion of different measurement approaches in research on diversity climate dimensions and facets to be essential, as a comprehensive understanding of diversity climate requires examining the phenomenon from multiple perspectives and by using multiple data sources. However, studies on diversity climate that incorporate multiple types of measures remain rare. Especially in light of our call to extend diversity climate research (e.g., by introducing additional facets), we see this as a timely opportunity to advocate for greater measurement pluralism and to outline concrete approaches that can support such efforts.

Most research on cultural diversity climate in schools has used surveys to assess students’ (and sometimes teachers’) perceptions of cultural diversity climate dimensions and facets (see Table 2 for sample items). Many survey studies on cultural diversity climate are cross-sectional, but researchers have also used surveys to map the development of cultural diversity climate over time (e.g., by employing yearly assessments) and to investigate longitudinal relations to outcome variables (e.g., Benner et al., 2008; Camacho et al., 2018; Karataş et al., 2023a). Surveys have clear strengths; for example, they are economical and easy-to-administer. Importantly, surveys allow researchers to gain insights into individuals’ subjective perceptions and interpretations of cultural diversity climate. Diversity climate may be perceived differently by different groups of students (e.g., cultural, ethnic, and racial majority vs. minority students), with minority students often perceiving lower levels of equal status (an optimal contact conditions facet), for example. Such variation in subjective experiences can only be captured by assessing students’ individual perceptions (Schachner, Schwarzenthal, & Noack, 2021).

Nonetheless, surveys also have their share of limitations. It may be difficult for students to retrospectively report on cultural diversity climate dimensions and facets present at their school. In addition, the common practice of assessing both cultural diversity climate and outcome variables using student self-reports introduces shared measurement methods and sources, which may inflate associations due to common method bias (Podsakoff et al., 2024). Such biases may, for example, stem from response biases such as social desirability or response patterns (e.g., acquiescent responding, Podsakoff et al., 2024; Röhl & Rollett, 2021).

Experience sampling, a particular application of surveys, can overcome some of the limitations of “classical” surveys (e.g., memory biases) and allows to address an intriguing set of questions on cultural diversity climate (see also Summary and Future Directions). In experience sampling, data is collected “in the moment” at specific points over time, often using very brief scales or single-item measures, while individuals are functioning within their natural environments, such as students being in school. However, experience sampling places heavy demands on participants, and is still subject to many of the same problems as regular surveys (e.g., social desirability, response patterns; Scollon et al., 2009).

Beyond surveys, several interesting measurement approaches exist that could be used to assess cultural diversity climate and advance theoretical understanding. These include methods such as classroom observations, coding of artifacts in both physical and virtual school environments, recording teachers’ talk in class, and analyzing school policy documents and digital behavioral trace data of interactions between students and between students and teachers (e.g., Celeste et al., 2019; Civitillo et al., 2017; Kehl et al., 2024; Walton et al., 2014). A common thread among these measurement approaches is that they provide a more objective^2^ measure of diversity climate compared to surveys, as they do not rely on the subjective perceptions of students or teachers. Additionally, they allow for the gauging of implicit cues about the diversity climate. For example, even if teachers do not report that they establish a colorblind climate (perhaps because they are unaware of it), their actual behavior in the classroom may tell a different story, which can be inferred from classroom observations, for example. Another advantage of many of the alternative measurements is that they avoid biases typically associated with self-reports, such as social desirability bias and response patterns.

However, the observation, recording, or extraction of cues believed to indicate dimensions and facets of cultural diversity climate does not necessarily mean that these cues are perceived in a similar way by students (e.g., Civitillo et al., 2017), which may limit their impact on students’ development and potentially lead researchers to draw misleading conclusions, such as inferring that a specific diversity climate dimension or facet is unrelated to student outcomes. With the hope of prompting researchers studying cultural diversity climate to embrace measurement pluralism to an even greater extent and combine different measures, we review the different measurement approaches in Table 4.

**Table 4.** Measurement of Cultural Diversity Climate Dimensions and Facets.

Measurement approach	Strengths	Limitations
(1) Surveys	- As surveys capture individuals’ subjective perceptions and interpretations of the cultural diversity climate, they can provide important insights - Surveys are cost-effective and easy to administer. They can be used to gather data from large numbers of students in many classes/schools - Student surveys can be utilized to offer teachers feedback on how students perceive specific dimensions and facets of the diversity climate within their class or school (e.g., Bijlsma & Röhl, 2023) - In addition to student ratings, teachers’ perceptions should also be captured with teacher ratings, as teachers play an important role in establishing diversity climates, and these two data sources can be triangulated	- Surveys come with several well-known limitations, including rating tendencies, retrospective bias, social desirability, and the risk of common method bias when both cultural diversity climate and outcome variables are assessed using student self-reports (e.g., Podsakoff et al., 2024). - Student and teacher ratings of cultural diversity climate typically show only weak agreement, and teacher ratings tend to show much smaller relationships and often no relationship with student outcome variables (e.g., Schwarzenthal et al., 2023)
(2) Experience sampling (ESM)/diary methods	- ESM, as a variation of surveys, can overcome some limitations of “classical” surveys (e.g., retrospective bias) because assessments are conducted in the moment - ESM assessments often take place in real-life situations, thus increasing ecological validity - ESM allows to map fine-grained temporal processes (e.g., Scollon et al., 2009), including within-person fluctuations in momentary perceptions of cultural diversity climate and their effects	- Several problems that pertain to surveys in general also apply to ESM (e.g., social desirability, cognitive biases). - ESM can be time-consuming and costly - Frequent assessments place a substantial burden on participants (Scollon et al., 2009)
(3) School policy documents	- Cultural diversity climate dimensions can be inferred from coding school policy documents consisting of mission statements, rules, and regulations regarding diversity (e.g., Celeste et al., 2019; Leer et al., 2025) - These documents provide a more objective measure of diversity climate than surveys	- The analysis of documents cannot provide insights into students’ and teachers’ perceptions and interpretations of cultural diversity climate - The existence of certain missions, rules, regulations does not ensure they are actually enacted at a school
(4) Artifacts in the physical and virtual school environment	- Artifacts in the physical and virtual school environment can be coded (e.g., posters on the school walls emphasizing the value of cultural diversity, forums, theater events with poems from students’ countries of origin, e.g., Civitillo et al., 2017) - Artifacts can capture more proximal school contextual characteristics indicative of cultural diversity climate dimensions and facets than school policy documents - Artifacts provide a more objective measure of diversity climate than surveys	- As for policy documents, the existence of certain artifacts (e.g., posters) does not necessarily imply that a specific diversity climate dimension or facet is actually present and perceived as such by students and teachers (Civitillo et al., 2017)
(5) Video observations/external observer ratings	- Classroom observations have been considered the “gold standard” for capturing teaching practices, as they provide a direct assessment of teaching practices - Observations can capture interactions in class from an outside perspective (e.g., Civitillo et al., 2017) - Videos provide a way to record and later analyze very detailed information (e.g., gestures, teacher gaze behavior)	- Such practices may be time-intensive and cost-intensive (e.g., video camera equipment, rater training) - Such practices may come with methodological problems (e.g., rater bias, measurement error, stability over time, Praetorius et al., 2014; White & Klette, 2023) - Videos and external observer ratings cannot provide insights into students’ and teachers’ subjective perceptions and interpretations of cultural diversity climate - Some cultural diversity climate dimensions and facets may be easier to observe (e.g., contact conditions: cooperation among students with different backgrounds; equal status) than others, thus potentially leading to the conclusion that the less observable ones are not present
(6) Recordings of teachers’ words	- The messages teachers convey in class can be indicative of the presence of specific cultural diversity climate dimensions (e.g., Walton et al., 2014) - If teachers carry small digital recording devices themselves, this measurement approach is much less time- or cost-intensive than video observations or external observations	- Without additional information (e.g., videos), it is challenging to include student data (student responses) and link student voices to specific students - Subtle information (e.g., gestures, teacher gaze behavior) cannot be captured - Voice data cannot provide insights into students’ and teachers’ subjective perceptions and interpretations of cultural diversity climate
(7) (Digital) traces of student–student and student–teacher interactions	- Analyses of behavioral trace data (e.g., from a school’s online learning platform, online discussion boards) can unobtrusively reveal fine-grained insights into interaction patterns that are potentially indicative of diversity climate dimensions and facets	- Students’ and teachers’ explicit subjective interpretations and perceptions cannot be captured - Cultural diversity climate may be more subtly conveyed in day-to-day interactions at school, and therefore might not become visible in trace data collected in a very specific (learning) context
(7) Interviews (and other qualitative approaches, e.g., focus groups)	- Interviews provide a way to gain in-depth insights into students’ and teachers’ interpretations and perceptions of cultural diversity climate dimensions and facets “in their own words”	- Interviews can be resource-intensive - Interviews are often conducted in small samples, and they are not designed to yield generalizable findings

*Note.* The focus here is on students and teachers, but surveys and other measurement approaches can of course also be used to gather data from principals and other school staff.

Different measurement and modeling approaches also (implicitly) convey different assumptions about the causality between measures of diversity climate and the diversity climate domains and facets. Within the classical latent variable framework, as applied in factor analysis, observed item responses (e.g., students’ ratings of multiculturalism climate) are assumed to be caused by underlying latent constructs (e.g., the multiculturalism climate in a school). Yet, there are also alternative ways of thinking about the causal relationship between diversity climate and its measures. For instance, if a school has a strong multiculturalism climate because its members actively celebrate group differences, then climate might be best conceptualized as an emergent property arising from shared behaviors and norms and could be modeled using network approaches (e.g., by using student ratings or trace data from online platforms). Conversely, if the celebration of diversity is considered to occur because the school has a strong multiculturalism climate, then climate operates more like a top-down contextual influence that shapes behaviors and interactions. In this case, researchers may rely more on measurements such as the coding of institutional policies or school leadership statements as presumed top-down indicators. We argue that there is no “right” or “wrong” perspective on these issues, as they depend on the underlying conceptualization of the relationship between diversity climate dimensions and facets and their respective measurements. Yet, we encourage researchers to be explicit about these assumptions.

## Constraints on Generality Statement

The conclusions presented in this article are informed by studies that include diverse student samples in terms of ethnicity, race, and cultural backgrounds, which is crucial given our focus on cultural diversity climate in schools. However, it is important to acknowledge that the majority of these studies are based on populations from the United States and Europe. While these regions provide valuable insights—especially given the challenges and opportunities related to increasing diversity and the ongoing issues of oppression and marginalization of certain groups—the geographic concentration limits the broader generalizability of our conclusions. Moreover, other dimensions of diversity, such as gender, socio-economic status, and their intersections with race, ethnicity, and cultural background(s), were typically not the primary focus of these studies. As a result, important blind spots remain that should be acknowledged when engaging with our work.

## Summary and Future Directions

This article provided an integrative, multidimensional, and multifaceted perspective on cultural diversity climate at school. We differentiated between cultural diversity climate and other related yet distinct concepts (e.g., diversity ideologies). An overview of the cultural diversity climate “Big Five,” with a focus on existing facets and newly introduced facets, and a discussion of their similarities and differences, was provided. We proposed an overarching metadimension matrix in which the different dimensions and facets can be located. In addition, measurement approaches were reviewed, outlining the advantages and pitfalls of each. Still, many questions remain, thus leaving room for intensified research efforts and more conceptual work. Therefore, we close the review with a 10-point plan that, from our perspective, generates the most needed and fruitful recommendations for guiding future investigations on cultural diversity climate.

*First*, we call for studies that address multiple dimensions and facets of cultural diversity climate to better understand its dimensional structure and the connections among different dimensions and facets, including potential interstitial facets (e.g., Bainbridge et al., 2022) and metadimensions. Of course, as a first step, the development and validation of measures for the newly introduced facets would be required. Here, researchers can take inspiration from the extensive work on the Big Five personality traits in personality psychology, where decades of study have resulted in refined and psychometrically sound measurement instruments of personality dimensions and facets, to develop a research agenda for the “Big Five” of cultural diversity climate and their facets.

It should be noted that some prior studies have investigated scales aligning with several of our dimensions and facets, and the results of their single-level factor analyses provided support for the proposed factor structure (Byrd, 2017; Schachner, Schwarzenthal, Moffitt, et al., 2021; for details on both studies, see Online Supplement S1). A comprehensive analysis of the factor structure of all dimensions and facets discussed in our article, ideally conducted at both the individual student and school or class levels using multilevel confirmatory factor analysis, has not yet been undertaken. However, we theorize that such research would reveal the same or a highly similar factor structure at the individual student and the classroom or school level. Cultural diversity climate is conceptualized as a group-level construct (i.e., a climate), with items typically phrased to reflect students’ shared classroom or school experiences (e.g., “In school, we are taught. . .”; “During class, we learn. . .”; Schachner, Schwarzenthal, Moffitt, et al., 2021). Therefore, the theoretically proposed dimensions and facets should emerge at the group level (i.e., classroom or school level). At the same time, we also emphasize the importance of individual students’ (idiosyncratic) perceptions of the diversity climate, which is why a multilevel approach that disentangles both levels of analysis is warranted. Complementing factor analyses, we further suggest employing psychometric network approaches to validate diversity climate scales (see, e.g., Christensen et al., 2020, for an example from personality psychology).

Ideally, future research should also triangulate different measurement approaches by combining traditional methods (such as surveys) with the various alternative assessment strategies outlined in this manuscript (e.g., observations, coding of artifacts and school policy documents, recordings of teachers’ talk; see Table 4). This is necessary to both broaden the scope of diversity climate research by moving beyond the strong focus on student-rated diversity climate, and to gain insights into the congruence between different measures of the same diversity climate construct. Except for a few studies that looked at the congruence between student and teacher ratings of diversity climate dimensions, knowledge on whether different measures may capture the same phenomena or tap into different constructs is largely absent. The studies on student–teacher congruence in their ratings document, at best, small to modest relationships, and higher levels of congruence for more easily observable dimensions (optimal contact conditions; e.g., whether group work and cooperation between student of different ethnic, racial, and cultural groups takes place in class) than less easily observable ones (multiculturalism climate; e.g., whether specific classroom practices indicate that cultural variation is valued; e.g., Civitillo et al., 2017). Both findings are in line with research on other school/classroom climate and teaching constructs (e.g., Wagner et al., 2016).

Furthermore, following standard practice in personality psychology research on the Big Five, we recommend that future research include all cultural diversity climate “Big Five” and their facets. Such an approach facilitates the reporting of null findings, better allows to compare findings across studies, and provides a more complete picture of which dimensions and facets are associated with a given outcome. Such an approach may also help disentangle the unique contributions of different, yet interconnected, dimensions and facets.

*Second*, robust empirical evidence of the temporal precedence of different cultural diversity climate dimensions and facets is still largely absent (for a notable exception, see Karataş et al., 2023a). It has been argued that the establishment of intergroup contact theory’s optimal contact conditions may form the basis for successfully implementing other dimensions; for instance, establishing basic preconditions (e.g., equal status of all students) may become a secure base from which to explore and learn about cultural diversity. In line with this, the study of Karataş et al. (2023a) demonstrated that contact conditions (scale focused on cooperation and positive contact) longitudinally predicted multiculturalism climate, whereas the effect in the other direction was not statistically significant. But do optimal contact conditions always come first, and to what extent do temporal influences vary across developmental periods and sociocultural contexts? For example, in highly unjust contexts, is it instead a critical consciousness climate that is needed to instigate changes and lead to other diversity climate dimensions (e.g., optimal contact conditions or multiculturalism climate; see also Dixon et al., 2010)? Another way to think about this issue may be to consider contact conditions as accompanying dimension that need to be in place not only to establish but also to maintain other dimensions and their positive effects; for example, learning about diversity as part of a multiculturalism climate in a classroom with a complete lack of optimal contact conditions (e.g., equal status, opportunities for students from different groups to work together) does not seem fruitful. However, even if contact conditions precede or accompany other dimensions, we still consider them as conceptually distinct, as their main theme clearly differs from that of the other diversity climate dimensions.

A further key question for future research concerns the temporal order of specific diversity climate facets. For example, regarding personal critical consciousness, Freire (1973, 2000) argued that critical reflection and critical action are reciprocally related. However, much research conceptualizes critical reflection as preceding critical action, with critical motivation as a mediator (Diemer & Rapa, 2016), or critical reflection and motivation as independent predictors of critical action (Hipolito-Delgado et al., 2024). Applied to diversity climate, it would be valuable to investigate whether promoting the critical motivation and reflection facets should come first, for example, before fostering the critical action facet.

*Third*, long-term effects of cultural diversity climate approaches, with studies spanning multiple years and educational levels, including potential carryover effects across contexts (e.g., the transition from school to university; carryover effects from the school context to out-of-school contexts, see Karataş et al., 2023b) are not well-understood. Relatedly, potential negative long-term side effects have not yet received systematic research attention. For instance, it has been proposed that positive intergroup contact can give rise to a sedative effect, such that members from ethnic, racial, and cultural minorities may feel less of a need to redress systemic inequities (Hässler et al., 2021). The extent to which such a sedative effect also occurs in schools with a strong focus on optimal contact conditions and harmonious intergroup relations needs to be explored.

*Fourth*, despite strong conceptual connections, there currently seems to be a scarcity of research linking cultural diversity climate dimensions at school with their “matching” diversity ideologies (e.g., multiculturalism climate and multiculturalism as a personal ideology) and other diversity ideologies. This situation stands in stark contrast to academic motivation-related school climate research, which often strongly centers on matching contextual and personal aspects of motivation (e.g., Bardach et al., 2020). In addition, future conceptual and empirical research building on our work may seek to further extend and refine the discussed facets ensuring they resonate even more with personal diversity climate ideologies. For example, an additional multiculturalism climate facet could focus on students’ learning about their multicultural society—an aspect that aligns with the personal multiculturalism ideology which centers on appreciating the contributions of different ethnic, racial, and cultural groups to society (Rosenthal & Levy, 2010).

*Fifth*, many psychologists and educational practitioners are most interested in the question of what dimensions and facets of cultural diversity climate are most likely to bring about change in desired outcome variables, and thus, in causality. Nonetheless, limited progress has been made in establishing causal effects in research on the cultural diversity climate at schools. A notable exception is the Identity Project intervention as there are some indications that the intervention could alter students’ cultural diversity climate perceptions (Schachner et al., 2024). Moreover, albeit the study by Bonam et al. (2019) did not focus on school students, they showed that a brief critical historical information intervention (which can be linked to the critical consciousness climate facet critical reflection) increased White adults’ acknowledgment of systemic racism (Salter et al., 2024). In addition to Randomized Controlled Trials, analyses of nonintervention longitudinal data that can at least better approximate causality (Bailey et al., 2018), including analytical techniques borrowed from other disciplines (e.g., economics research), offer worthwhile future research directions.

*Sixth*, the dynamic nature of students’ perceptions of cultural diversity climate approaches at school should be placed at the center of future research endeavors. For instance, the same student likely experiences the presence of different dimensions and facets over time and even within 1 school day to varying extents (see Figure 2). Interindividual differences in these intraindividual trajectories should emerge as well, in that some students may be more susceptible to certain messages or may be more likely to experience certain aspects of cultural diversity climate, depending on their background and history. Further, dynamic interactions (e.g., between students and teachers, including differential teacher treatment) may come into play and should be accounted for. Experience sampling methods (e.g., Yip et al., 2020; Yip & Douglass, 2013, see also Table 4) hold promise for better capturing such dynamics, whereas future diversity climate theory development should be enriched by considering dynamic diversity climate “states” (see Jayawickreme et al., 2021, for an overview of how dynamic processes have been integrated into the definition and assessment of personality).

**Figure 2.**
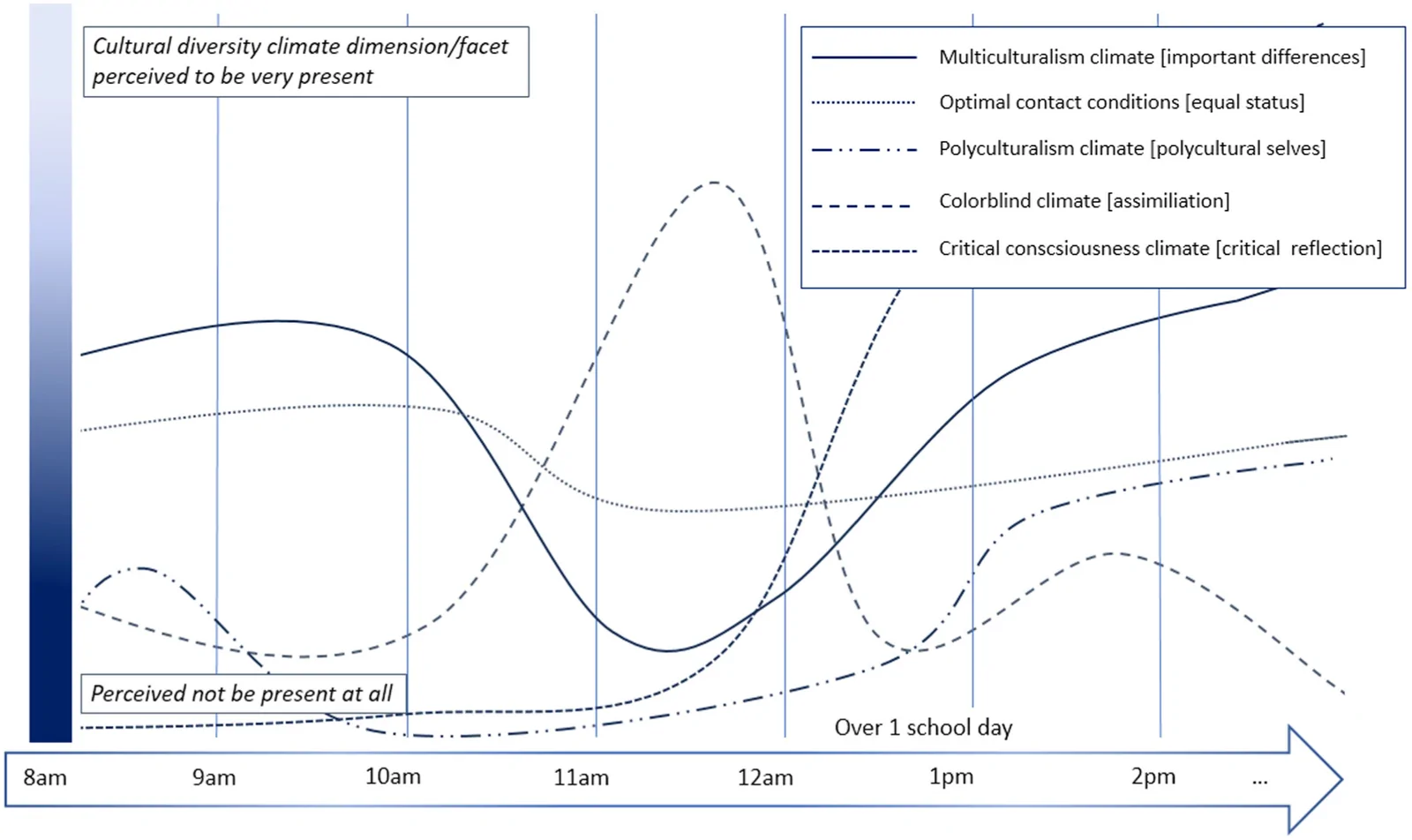
Cultural diversity climate dynamics. *Note.* Example of how one’s students’ state-level perceptions of five facets of five cultural diversity climate dimensions could shift over the course of one school day.

*Seventh*, research on cultural diversity climate should be diversified, in terms of both samples (given that most current research stems from the United States and Europe, see also Constraints on Generality Statement) and research settings. Diversifying research samples is crucial because the sociocultural context can strongly impact the manifestation, interpretation, and effects of different diversity climate dimensions and facets (for similar arguments regarding parental ethnic–racial socialization, see e.g., Aral et al., 2021). Currently, we do not know whether insights from research on cultural diversity climate derived from populations in the United States and Europe apply to other countries and cultural contexts. Specifically, we largely lack insights into how different cultural diversity climate dimensions and facets manifest in majority world countries (i.e., countries in which most of the world’s population lives, i.e., Africa, Asia, Central and South America, and the Middle East, see e.g., Alam, 2019; Khan et al., 2022; United Nations, 2019). This issue also has implications for cultural diversity climate measures, which may need to be adapted or even newly developed to better account for contextual specificities. Additionally, when using the same cultural diversity climate measures in different contexts, non-invariant scales and items may provide an opportunity (rather than a nuisance) to detect fundamental cultural differences in perceptions of cultural diversity climate (see Achaa-Amankwaa et al., 2021, who make this argument in personality psychology). In fact, research on cross-cultural psychology has long argued for the combination of emic (culture-specific) and etic (culture-comparable) approaches (e.g., Cheung et al., 2011). Future research should also more fully engage with other critical dimensions of diversity, such as gender and socio-economic status, and importantly, their intersections with race, ethnicity, and cultural background (e.g., Crenshaw, 1991; Keller et al., 2023). Attending to these intersections is essential for capturing the complex and layered nature of individuals’ experiences and the contextual affordances that shape them.

The diversification of research settings can be achieved in many ways. Here, we discuss two potential avenues. For example, experimental research has demonstrated that playing specific videogames may reduce prejudices and stereotypes (see, e.g., the finding that playing the videogame “PeaceMaker” for 20 min reduced explicit stereotypes; Alhabash & Wise, 2015), and effects of indirect (virtual) intergroup contact and optimal contact conditions are well-established (see, e.g., online contact involving a preprogrammed outgroup member; White et al., 2021). Future research in this realm could adopt educationally relevant tools (e.g., in the form of serious games, i.e., games developed for educational purposes, Deterding et al., 2011) that include cultural diversity climate dimensions beyond optimal contact conditions. To illustrate, a serious game for history classes in secondary school in which students learn about the history of the Roman Empire, the relationships between the Romans and Barbarian tribes, and influences between different cultures, could be designed so that it includes cultural diversity climate-related messages (e.g., polyculturalism climate; Viccari et al., 2024). Another avenue for future psychological research may involve exploiting the potential of virtual reality, which can combine experimental control with authentic learning settings (e.g., Huang et al., 2021). Hence, in a virtual reality classroom, the behavior and talk of virtual classmates and/or a virtual teacher—and thus, different cultural diversity climate dimensions and facets—could be experimentally manipulated to investigate their effects on outcomes (also linked to the fifth recommendation focusing on causality).

*Eighth*, inequities and systems of oppression are prevalent in many societies (Heberle, Rapa, & Farago, 2020: Salter et al., 2018). It is thus most pressing to conduct intensified research on cultural diversity climate dimensions that foreground or deny such issues (i.e., critical consciousness climate and colorblind climate). We already know that optimal contact conditions promote positive intergroup contact and reduce prejudice (e.g., Pettigrew & Tropp, 2006; Tropp et al., 2022). What we now need to better understand are the structure and effects of other dimensions directly tied to social inequities. Relatedly, future research in this realm would do well to account for intersecting social categories (Heberle, Obus, & Gray, 2020; Lei et al., 2023; McKellar et al., 2024).

*Ninth*, research on diversity climate at school can both inspire other areas and take inspiration from them. For instance, the differentiated and fine-grained facet-level conceptualization put forth in this review may offer new ways of approaching cultural diversity climate in other social contexts and research areas. On the other hand, research on cultural diversity climate can be enriched by considering insights from other areas. Research in work and organizational psychology, for example, has long complemented its focus on mean levels of climate constructs with an interest in within-group agreement on these constructs, an approach that can also be applied to studying cultural diversity climate in schools. Cultural diversity climate researchers can also gain valuable insights from the parental ethnic–racial socialization literature (e.g., Hughes et al., 2006; Wang et al., 2020; Saleem & Byrd, 2021). For instance, research on parental socialization often distinguishes between implicit socialization (e.g., the number of outgroup friends parents have) and explicit socialization (e.g., direct messages). It may be beneficial to explore the implicit–explicit continuum more deeply in the context of cultural diversity climate research. Saleem and Byrd (2021) discuss how diversity climate-related messages can be conveyed at school by drawing on ideas from the parental ethnic–racial socialization literature. These messages could be distinguished on the two dimensions of expression (which can further be divided in verbal and non-verbal messages), and intent in terms of the purpose of the message (active message with a purpose; passive messaging with an unintended purpose; see also Lesane-Brown, 2006; Yasui, 2015). Additionally, parental ethnic–racial socialization is theoretically considered a bidirectional process (Hughes et al., 2006), and recent studies highlight adolescent-directed patterns of ethnic–racial socialization (e.g., Patel et al., 2023; Schwarzenthal et al., 2025). Therefore, it could be worth investigating whether teachers are always the primary influencers of diversity climate, or if adolescents—who possess various cultural affiliations and learn about social inequity and racism through social media, among other sources—also play an active role in shaping the diversity climate “bottom-up.” However, an important consideration is that schools are often structured in a hierarchical manner, which may limit the extent to which youth-directed socialization processes can occur. Lastly, researchers in ethnic–racial socialization have increasingly emphasized the importance of an ecological approach, exploring how ethnic–racial socialization occurs across different contexts and how these contexts interact (e.g., Aral et al., 2024; Hughes et al., 2016; Sladek et al., 2022). Therefore, a promising avenue for future research could be to examine the interplay between school cultural diversity climate and socialization within parent and peer contexts (see also Kornienko et al., 2024; Miklikowska et al., 2019).

*Tenth*, we have introduced the five cultural diversity climate dimensions and their facets separately for conceptual clarity. However, the different dimensions and facets likely co-occur in educational settings, and it has even been suggested that different dimensions are best combined to achieve the most positive effects (Schwarzenthal et al., 2022). Future research adopting person-centered approaches is needed to examine configurations of perceived diversity climate dimensions and facets within individuals (e.g., Hernández et al., 2025). Such research would, for example, allow to disentangle the effects of belonging to a profile characterized by high levels of contact conditions and colorblind climate, as compared to belonging to a profile characterized by the combination of high levels of contact conditions and multiculturalism climate. While we assume that the former should be maladaptive (e.g., for the development of minority students’ ethnic–racial identity), students in the latter profile may be able to exploit the benefits of both contact conditions and of multiculturalism climate, which addresses blind spots of contact conditions. Longitudinal approaches, including the study of transitions between profiles, constitute important further steps. Moreover, profiles can also be modelled within a multilevel framework; for example, by focusing on individual student profiles at the individual student level, which are then used to identify latent classes at the class level based on the relative frequencies of individual student level profiles within these classes (Mäkikangas et al., 2018; see also Bardach et al., 2022). In this way, we can comprehensively characterize entire classroom or school environments (e.g., classes with a large proportion of students belonging to profiles that strongly feature specific colorblind climate facets; classes including students whose profiles mostly suggest an emphasis on critical consciousness climate and multiculturalism climate, with some indications of the perceived presence of polyculturalism climate). Relatedly, thinking about the systematic combination of dimensions raises the important question of for whom and under which circumstances should which dimensions and facets be jointly addressed, and how educators can be best prepared to do so (see also research on personalized learning/adaptive teaching, e.g., Dumont & Ready, 2023).

## Conclusions

Schools play a key role in shaping how students experience and understand cultural diversity. This review presented the cultural diversity climate “Big Five” framework and emphasized the need for a more fine-grained understanding by further developing existing facets and introducing new, theory-driven ones. Considering the five dimensions and their 17 facets together provides deeper insights for advancing psychological theory on diversity climate than examining them in isolation, in pairs, or at the dimension level without attention to their facets. For instance, the inclusion of all dimensions and facets within a single framework makes it possible to theorize their interconnectedness, and thus, how one may amplify, buffer, or even compensate for the shortcomings of another. Furthermore, our framework allows for theorizing on and exploring the developmental interplay and temporal precedence of dimensions and facets, thus strengthening the theoretical foundation of diversity climate research. Also, it allows to examine meaningful profiles that would remain obscured when each dimension is studied in isolation and that may carry unique psychological implications for student outcomes such as belonging, academic motivation, or intergroup attitudes. Importantly, prior work on diversity climate dimensions has lacked systematic facet-level integration; hence, the facet level introduced here may provide, akin to personality facets, more comprehensive descriptions, potentially more accurate predictions of critical outcome variables, and a better understanding of the mechanisms underlying diversity climate dimension–outcome relationships (Danner et al., 2021). Another novel contribution of our work is the proposal of an overarching metadimension matrix in which the different dimensions and facets can be located.

Taken together, we believe that an integrative, multidimensional, and multifaceted diversity climate framework has much to offer for building a psychological theory of diversity climate that accounts for real-world complexity—after all, cultural diversity climate remains a complex, real-life phenomenon. Therefore, it is both a crucial challenge and an opportunity for psychological research on cultural diversity climate to increase its potential impact on practice and policy (see also, e.g., Paluck et al., 2019; Phalet & Baysu, 2020), with the overarching aim of improving the educational experiences and life chances of children and adolescents growing up in diverse and often unequal worlds.

## Supplemental Material

sj-docx-1-psr-10.1177_10888683261458924 – Supplemental material for The Cultural Diversity Climate “Big Five” in Schools: Dimensions, Facets, and a Future Research Agenda Supplemental material, sj-docx-1-psr-10.1177_10888683261458924 for The Cultural Diversity Climate “Big Five” in Schools: Dimensions, Facets, and a Future Research Agenda by Lisa Bardach and Miriam Schwarzenthal in Personality and Social Psychology Review

## Notes

1. While both the DIAMONDS taxonomy and cultural diversity climate dimensions address characteristics of situations/contexts, they differ in scope, specificity, and conceptual focus. The DIAMONDS dimensions represent broad and abstract situational characteristics intended to describe a wide range of everyday situational experiences not tied to any specific social construct (e.g., Duty: “A job needs to be done”; Mating: “Potential romantic partners are present,” Rauthmann et al., 2014). In contrast, diversity climate dimensions are more narrowly defined, capturing aspects related to ethnic, racial, and cultural diversity within specific social environments, such as schools, and often in reference to particular others within these settings, such as teachers and peers. Additionally, diversity climate tends to focus on collective environments, such as classrooms or schools, and can therefore be conceptualized as a phenomenon operating at both the individual and group levels (e.g., class or school). In contrast, the DIAMONDS dimensions typically describe psychological features of situations as experienced by individuals.

2. We use the term objective measures in line with common terminology in the literature to refer to externally assessed indicators, as distinct from students’ and teachers’ self-reported perceptions. However, because these measures still involve human interpretation (e.g., the coding of videos), we acknowledge that some scholars prefer the term intersubjective.
