# Supplementary material for: The Cultural Diversity Climate “Big Five” in Schools: Dimensions, Facets, and a Future Research Agenda
Source: Pers Soc Psychol Rev. 2026 Jul 3;30(4):609–35. doi: 10.1177/10888683261458924 (PMC13672948; doi:10.1177/10888683261458924)
Supplement: Supplementary file 1 [file sj-docx-1-psr-10.1177_10888683261458924.docx]

**Online Supplement S1: Prior factor-analytic work**

Byrd (2017) examined the factor structure of scales aligning with several of our dimensions and facets in two samples of middle and high school students from the U.S., with equal proportions of Black, White, Asian American, and Latino/a students. Specifically, the study included intergroup contact theory’s optimal contact conditions—equal status, association (referred to as quality of interaction), a scale focusing primarily on cooperation (referred to as frequency of interaction), and authority support (referred to as support for positive interaction). It also addressed two multiculturalism climate facets: being affirmed in one’s own culture (referred to as cultural socialization) and important differences (referred to as promotion of cultural competence). In addition, the study included a critical consciousness climate scale (aligned with the critical reflection facet introduced in the current manuscript and referred to as critical consciousness socialization) and a colorblind climate scale (aligned with the ignoring differences facet and referred to as colorblind socialization). Byrd (2017) reported the results of a single level confirmatory factor analysis based on these dimensions, as well as some others not included in our work (e.g., stereotyping). A good level of fit to the data was documented in both studies. The reported standardized factor loadings ranged from .46 to .85 (Study 1), and from .51 to .86 (Study 2).

In another study, dimensions corresponding to several of those described in our work were investigated in a sample of German secondary school students using single-level exploratory and confirmatory factor analyses (Schachner et al., 2021a). The six dimensions identified in the factor analyses included a contact conditions scale combining association and cooperation (referred to as contact and cooperation); a contact conditions scale focusing on equal status (referred to as unequal status); a colorblindness scale capturing the similarities facet of a colorblind climate (referred to as color-evasion); a scale combining the two multiculturalism climate facets discussed in our manuscript (referred to as heritage culture and intercultural learning); a critical consciousness climate scale focusing on the critical reflection facet described in our manuscript; and a polyculturalism climate scale combining the two facets of polyculturalism climate discussed in our manuscript (intercultural influences, polycultural selves). The models fit the data well, and standardized factor loadings ranged between .63 and .94. The authors also conducted extensive measurement invariance testing, showing that the scales were invariant (scalar invariance) across gender, school track, and migration background.
